# Electrochemiluminescence/Electrochemistry Dual-Mode Synchronous Sensing of Pb^2+^ Based on G4–hemin DNAzyme Complex During One-Step Scan

**DOI:** 10.3390/molecules30091951

**Published:** 2025-04-28

**Authors:** Rukai Wei, Lei Shang, Wei Zhang, Xiaojian Li, Liping Jia, Rongna Ma, Huaisheng Wang

**Affiliations:** Department of Chemistry, Liaocheng University, Liaocheng 252059, China

**Keywords:** dual mode, ECL/EC, one-step scan, G4–hemin, Pb^2+^

## Abstract

Electrochemiluminescence (ECL)/electrochemistry (EC) dual-mode sensors have garnered significant interest for their enhanced analytical reliability through the cross-verification of dual-signal outputs. However, conventional approaches necessitate two potential scans to acquire ECL and EC signals independently, resulting in temporal and environmental discrepancies between the two detection modes. In this paper, we present a novel synchronous ECL/EC dual-mode sensing platform for lead ion (Pb^2+^) detection via a one-step potential scan (0.2 to −0.4 V vs. Ag/AgCl) utilizing a G-quadruplex (G4)–hemin DNAzyme complex. This complex synergistically catalyzed the electrochemical reduction of dissolved oxygen, concurrently generating a distinct cathodic ECL emission from Ru(bpy)_3_^2+^ and a synchronous reduction current peak at −0.25 V. Pb^2+^ quantification was achieved through its dose-dependent suppression of DNAzyme activity by destabilizing the G4–hemin interaction, thereby proportionally attenuating both ECL intensity and EC signal (reduction current). The integrated sensor demonstrated high sensitivity (detection limits of 1.51 nM for ECL detection and 2.03 nM for EC detection), robust anti-interference capability, and satisfactory reproducibility, with recoveries ranging from 95.5 to 103.1% in environmental water analysis. This work established a paradigm for one-step dual-mode sensor design, offering new prospects for environmental monitoring.

## 1. Introduction

Lead ions (Pb^2+^), a persistent and bioaccumulative environmental toxin, pose profound and irreversible health consequences for humans [1,2,3]. Thus, it is crucial to develop highly sensitive and accurate methods for detecting Pb^2+^ in the environment. While conventional methods such as atomic absorption spectroscopy (AAS) [4], inductively coupled plasma mass spectrometry (ICP-MS) [5], colorimetry [6,7], and fluorescence (FL) techniques [8] offer high sensitivity and accuracy, their reliance on lab-bound instrumentation, high costs, and complex protocols hinders on-site application. As a powerful analytical technology, electrochemiluminescence (ECL) could achieve a fast, sensitive, and selective detection of Pb^2+^ [9,10]. For example, an ultrasensitive “on–off” ECL biosensor based on MXene@Au and Au@SiO_2_ nanoparticles was developed to offer a novel platform for trace Pb^2+^ detection [11]. However, the detection based on single ECL is inevitably affected by the disturbance of instruments and the complex test environments.

ECL-based dual-mode sensing, such as ECL/fluorescence [12,13], ECL/colorimetry [10,14], ECL/electrochemical impedance spectroscopy (EIS) [15,16], ECL/electrochemistry (EC) [17,18], ECL/photoelectrochemistry (PEC) [19,20], and ECL/surface-enhanced Raman scattering (SERS) [21], can offer improved detection reliability by leveraging internal self-calibration between ECL and the other signal to minimize interference from environmental variables. Among these, the ECL/EC strategy has garnered significant interest in the detection of Pb^2+^ due to its low cost, ease of operation, and ability to generate both ECL and EC signals using a single electrochemical platform. For example, Hu et al. constructed a dual-mode ECL/square wave voltammetry (SWV) sensor of Pb^2+^ based on ECL signals (0–1.5 V vs. Ag/AgCl) from Ru(phen)_3_Cl_2_ and SWV response (−0.8 to −0.4 V vs. Ag/AgCl) from stacked crystal violet [22]. Another sensor was used for the ECL/fast scan voltammetry (FSV) dual-mode determination of Pb^2+^ by providing an EC signal of ferrocene from −1.0 to +1.0 V at a scan rate of 100 V s^−1^ and an ECL signal of Ru-NH_2_ from +1.0 to +1.8 V with a scan rate of 0.1 V s^−1^ [23]. However, although sensitive ECL/EC dual-mode detections of Pb^2+^ have been achieved, there are still some problems: (1) two independent scans in different potential ranges are needed to obtain ECL and EC signals; (2) the distant applied potentials lead to different test times and environments between ECL and EC detection; and (3) the excess triggered ECL potential causes the electrical destruction of biomolecules. These problems influence the detection accuracy.

**Scheme 1:**
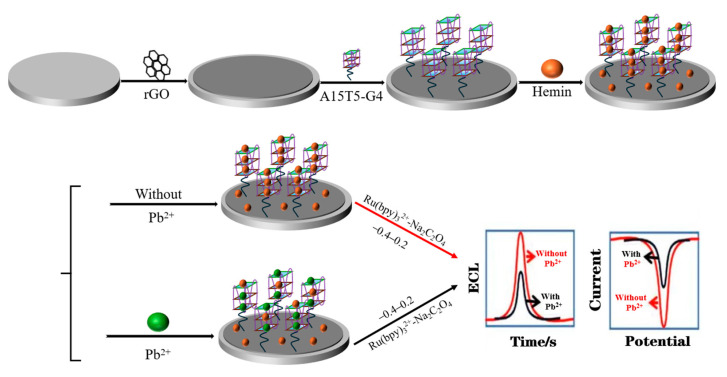
The process of constructing the dual-mode ECL/EC sensor.

In this work, we built an ECL/EC dual-mode sensing platform capable of simultaneously performing ECL and EC detection during a one-step scan in a low-potential range. As illustrated in Scheme 1, reduced graphene oxide (rGO) was first drop-cast onto a clean glassy carbon electrode (GCE) surface to form GCE/rGO. Subsequently, an unmodified G-quadruplex with an A15T5 head (A15T5-G4) was immobilized onto the GCE/rGO surface via strong π–π stacking interactions between A15T5 and rGO [24,25]. Finally, with the assistance of K^+^, hemin was intercalated into G4 to form the G4–hemin DNAzyme complex [26]. During a one-step scan from 0.2 to −0.4 V, the obtained rGO/G4–hemin hybrid possessed excellent activity toward the electrochemical reduction of oxygen in the generation of hydroxyl radicals (HO·), which could trigger Ru(bpy)_3_^2+^ to produce a strong cathodic ECL signal employing Na_2_C_2_O_4_ as a coreactant, so a distinct ECL peak and reduction current peak synchronously appeared at −0.25 V. In the presence of Pb^2+^, it could reduce the affinity between G4 and hemin [27] and cause hemin to move away from the electrode surface, leading to the synchronous reduction in current and the cathodic ECL signal. Notably, the detection could be achieved during a one-step scan from 0.2 to −0.4 V, and the applied low potential range effectively protected the biomolecules from electron-induced damage, thereby enhancing the reliability of the detection method.

## 2. Results and Discussion

### 2.1. Characterization of Electrode Materials

**Figure 1:**
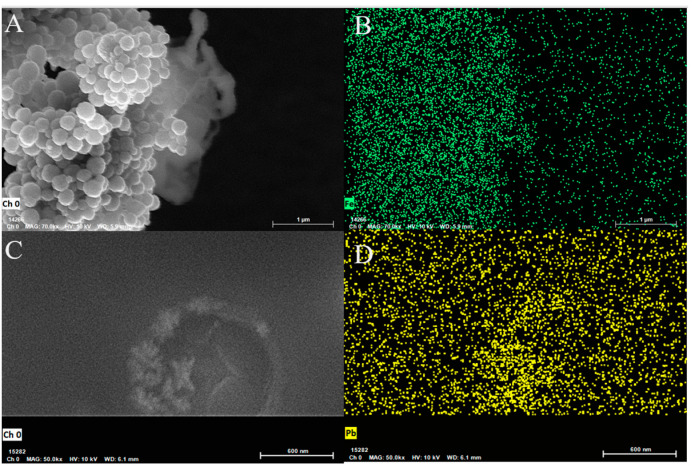
SEM images of rGO/G4–hemin (**A**) and rGO/G4–hemin/Pb^2+^ (**C**) along with their corresponding EDS mapping images (**B**,**D**).

In this study, rGO was successfully synthesized by reducing GO with sodium borohydride (NaBH_4_) [28], which was confirmed by UV-Vis characterization [29]. After reduction, the product displayed a single absorption peak at approximately 270 nm, suggesting a decrease in C=O groups while preserving the conjugated system (Figure 1B). This result confirmed the formation of rGO. With the assistance of K^+^, G4 formed a folded structure, which had a weak interaction with rGO [30,31]. Thus, in order to anchor G4 onto the rGO, we added A15T5 on the head of G4 because polyadenine strongly interacts with rGO [24,25]. After hemin was incubated on rGO/A15T5-G4, a scanning electron microscopy (SEM) image showed that many particles were produced, and the EDS-mapping characterization showed that these particles contained Fe elements (Figure 1A,B). This result revealed that the hemin was embedded in G4 and aggregated into nanoparticles, forming an rGO/A15T5-G4/hemin hybrid. After Pb^2+^ was added, the nanoparticles reduced (Figure 1C and the Pb element appeared (Figure 1D), because Pb^2+^ could bind with G4, decreasing the binding affinity of hemin with G4 to release hemin away from G4 [32].

### 2.2. Electrochemical Characterization of the Dual-Mode Sensor

**Figure 2:**
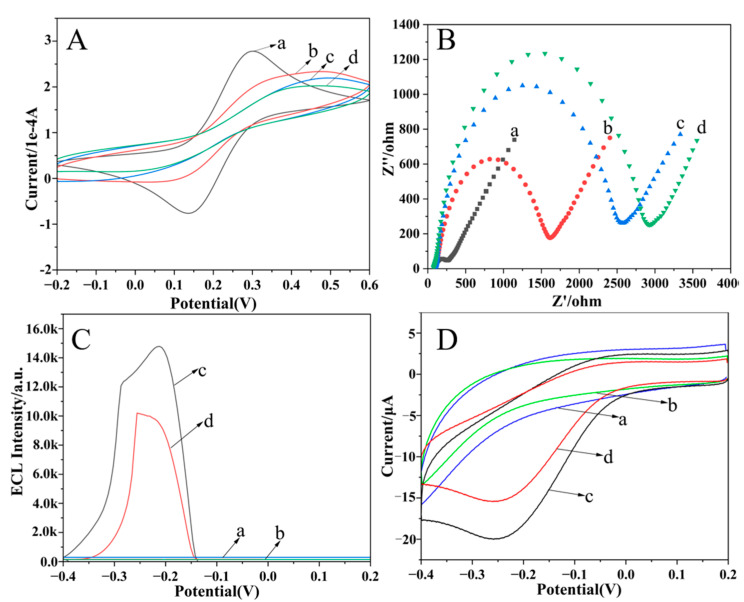
CV (**A**) and EIS (**B**) curves of GCE/rGO (a), GCE/rGO/A15T5-G4 (b), GCE/rGO/G4–hemin (c), and GCE/rGO/G4–hemin/Pb^2+^ (d) in a 0.1 M KCl solution containing 10 mM [Fe(CN)_6_]^3−/4−^. ECL-potential (**C**) and CV (**D**) curves of GCE/rGO (a), GCE/rGO/A15T5-G4 (b), GCE/rGO/G4–hemin (c), and GCE/rGO/G4–hemin/Pb^2+^ (d) in an air-saturated Ru(bpy)_3_^2+^–Na_2_C_2_O_4_ system. Scan rate: 0.1 V/s; PMT: 650 V; frequency in EIS: 0.01–10^5^ Hz.

Cyclic voltammetry (CV) and electrochemical impedance spectroscopy (EIS) effectively reflect the condition of the electrode surface. Therefore, CV characterization was first conducted to characterize the sensor fabrication process (Figure 2A). The GCE/rGO exhibited a pronounced redox peak current owing to the well-conducing rGO, which accelerated the electron transfer of [Fe(CN)_6_]^3−/4−^ at the electrode interface. Subsequently, the immobilization of the G4 strand caused a decreased redox peak current attributed to its electrostatic repulsion toward [Fe(CN)_6_]^3−/4−^. After hemin and Pb^2+^ were incubated, the redox peak currents continued to drop to almost the same level, attributed to the steric hindrance effects and blocking the electron transfer channel from the stable G4–hemin or G4-Pb^2+^ complex. Subsequently, electrochemical impedance spectroscopy (EIS) was performed (Figure 2B). The electron transfer resistance (*R_et_*) of the GCE/rGO electrode was relatively low. When the A15T5-G4 strand was immobilized on the electrode, *R_et_* distinctly increased. The successive incubation of hemin and Pb^2+^ made *R_et_* further increase. The variation tendency was akin to the previous report [33], and the consistency between the EIS and CV results confirms the successful execution of the sensor fabrication.

### 2.3. Feasibility of Dual-Mode Sensor for Pb^2+^ Detection

Furthermore, the feasibility of the developed dual-mode ECL/EC sensor was estimated by recording the ECL and EC behaviors on different modified electrodes in an air-saturated Ru(bpy)_3_^2+^–Na_2_C_2_O_4_ system. As shown in Figure 2C,D, neither the GCE/rGO nor the GCE/rGO/A15T5-G4 electrodes exhibited ECL signals or redox current peaks. However, when hemin interacted with G4 to form the G4–hemin DNAzyme complex, distinct ECL and a reduction current peak were synchronously observed at −0.25 V (vs. Ag/AgCl) during a one-step scan from 0.2 to −0.4 V, indicating that the G4–hemin DNAzyme complex was necessary to produce the synchronous ECL and EC signals. After Pb^2+^ was introduced to the GCE/rGO/G4–hemin electrode, the ECL and EC signals were both markedly diminished. This was because Pb^2+^ could reduce the affinity between G4 and hemin [27,32] and cause hemin to move away from the electrode surface. This result confirmed the successful ECL/EC dual-mode detection of Pb^2+^ during a one-step scan. Notably, both the ECL and reduction peak potentials remained at the relatively low level of −0.25 V, which helps prevent electron-induced damage to the biomolecules, thereby enhancing the detection stability and reliability. Based on these results, the synergistic interplay between the ECL and EC signals enabled the development of a high-precision dual-mode ECL/EC sensor.

### 2.4. Generating Mechanism of ECL and EC Signals

**Figure 3:**
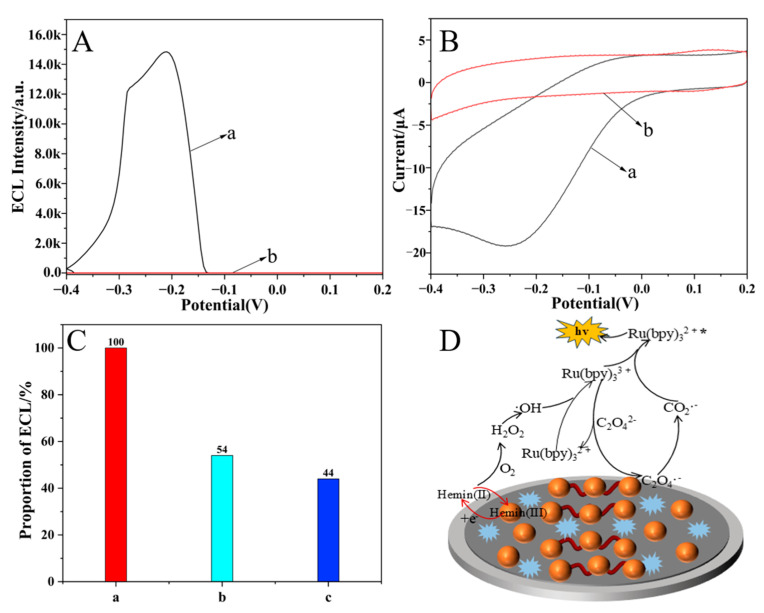
ECL–potential (**A**) and CV (**B**) curves of GCE/rGO/Apt/Hemin in an air-saturated (a) and nitrogen-saturated (b) Ru(bpy)_3_^2+^–Na_2_C_2_O_4_ system. (**C**) The ECL percentage in an air-saturated Ru(bpy)_3_^2+^–Na_2_C_2_O_4_ system for the blank (a) and in the presence of 10 nM DA (b) and 10 nM AA (c). (**D**) The mechanism of cathodic ECL generation.

To elucidate the generating mechanism of the ECL and EC signals, we conducted a series of investigations. Based on [34], it is reasonably hypothesized that the ECL and EC signals are closely associated with the presence of dissolved oxygen in the solution. To test this hypothesis, nitrogen was bubbled into the air-saturated Ru(bpy)_3_^2+^–Na_2_C_2_O_4_ system to remove oxygen. As can be seen in Figure 3A,B, ECL and current signals were barely observed on the GCE/rGO/G4–hemin electrode. Upon reintroducing air into the electrolyte, distinct ECL and reduction current signals reappeared, indicating that the reduction current peak resulted from the oxygen reduction reaction (ORR) and the ECL signal was related to the ORR. Inspired by the previous report on a cathodic ECL in Ru(bpy)_3_^2+^–TPA [34], we inferred that the ECL signal was also triggered by ·OH during ORR. To verify this deduction, dopamine (DA) and ascorbic acid (AA), both possessing OH scavenging properties, were introduced into an air-saturated Ru(bpy)_3_^2+^–Na_2_C_2_O_4_ electrolyte. The results demonstrated that the ECL intensity decreased to 54 and 44% of its original value, respectively (Figure 3C). These findings indicated that the ECL was derived from the ORR, thereby guaranteeing the synchronization of the ECL and EC signals during a one-step scan. Based on the aforementioned results, the potential mechanism of inducing ECL and reduction current is illustrated in Figure 3D along with the following reaction equations:

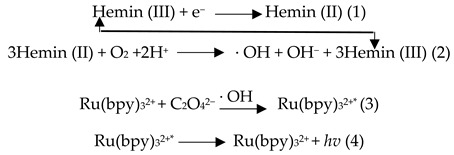


As a result of the ORR catalyzed by the G4–hemin complex (Equations (1) and (2)), a pronounced reduction current was observed. Subsequently, the generated ·OH radicals initiated a cathodic ECL response in the Ru(bpy)_3_^2+^–Na_2_C_2_O_4_ system, as illustrated in Equations (3) and (4). In summary, both hemin and dissolved oxygen are indispensable for the generation of ECL signals and the associated reduction peak currents in this system.

### 2.5. Optimization of Experimental Conditions

**Figure 4:**
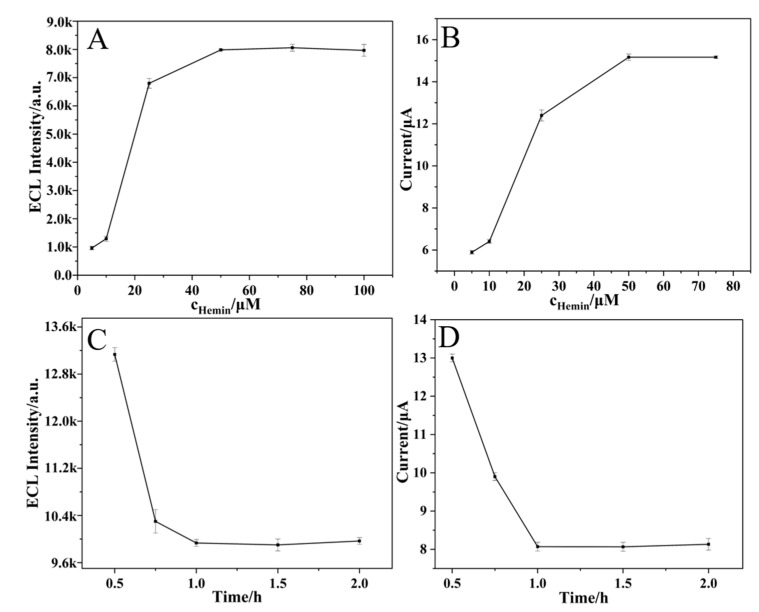
Effect of hemin concentrations on ECL (**A**) and EC (**B**) signals. (**C**) Effect of incubation time of Pb^2+^ on ECL (**C**) and EC (**D**) signals.

As mentioned above, the amounts of hemin and Pb^2+^ could affect the ECL and EC signals, so the hemin concentration and Pb^2+^ incubation time were optimized. With increasing hemin concentration, both ECL intensity and reduction peak current increased until reaching a maximum at 50 μM (Figure 4A,B). Moreover, these two signals gradually decreased with longer Pb^2+^ incubation times and stabilized after 1 h (Figure 4C,D). Consequently, 50 μM of hemin and 1 h of incubation time of Pb^2+^ were selected.

### 2.6. Detection of Pb^2+^

**Figure 5:**
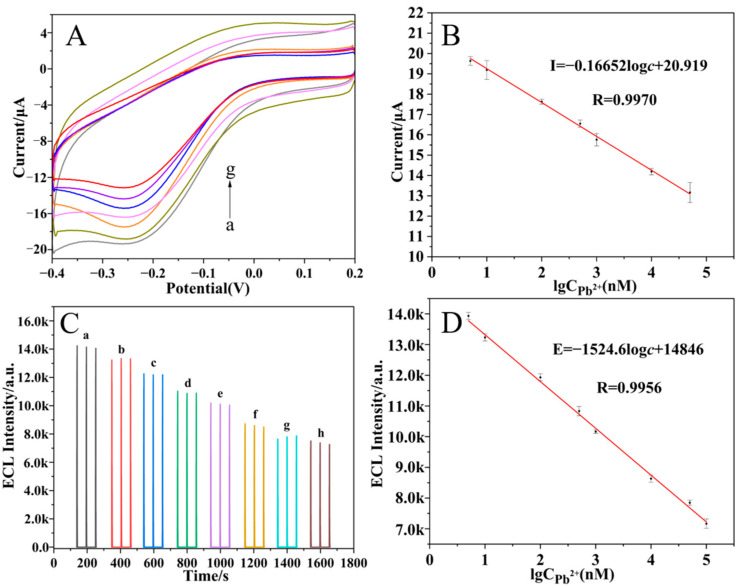
(**A**) Current–potential curves for different concentrations of Pb^2+^. (**B**) Linear correlation between current and log*c*: (a→g: 5, 10, 100, 500, 10^3^, 10^4^, and 5 × 10^4^ nM). (**C**) ECL–time curves for different concentrations of Pb^2+^. (**D**) Linear correlation between ECL intensity and log*c*: (a→h: 5, 10, 100, 500, 10^3^, 10^4^, 5 × 10^4^, and 1 × 10^5^ nM).

Under optimized experimental conditions, the performance of the ECL/EC dual-mode sensor was evaluated. The reduction peak current (*I*) and the ECL intensity (*E*) both gradually declined with increasing Pb^2+^ concentration, and two linear regression equations for *I* and *E* were, respectively, determined to be *I* = −0.16652 log *c* + 20.919 (*c* corresponds to the Pb^2+^ concentration within 5–50 μM) with a correlation coefficient (R) of 0.9979 and a detection limit (LOD) of 2.03 nM (S/N = 3) (Figure 5A,B), and *E* = −1524.6 log *c* + 14846 (*c* is the Pb^2+^ concentration in the range of 5–100 μM) with an R value of 0.9975 and an LOD of 1.51 nM (S/N = 3) (Figure 5C,D). Compared with a previous report on Pb^2+^ detection (Table 1), the developed dual-mode sensor showed superior performance on the detection limit and the linear range. More importantly, the mutual calibration of the two signals enhanced the overall detection accuracy compared with these single-signal-based sensors.

Furthermore, compared with other studies on ECL/EC dual-mode sensors with different ECL and EC potential ranges (Table 2), the dual-mode sensor developed in this work simultaneously generated both ECL and EC signals at a potential of −0.25 V, thereby ensuring a consistent detection environment during a one-step scan. Meanwhile, the peak potential was low enough to effectively safeguard the electrode and biomolecules against electronic damage, collectively enhancing the reliability and accuracy of the measurements. Moreover, we noted that Wang et al. [40] also employed the G4–hemin composite structure for the detection of Pb^2+^ with a lower detection limit by rolling circle amplification. Inspired by this result, we planned to integrate this signal amplification technique into our future dual-mode sensor, aiming to obtain high sensitivity while maintaining signal calibration.

### 2.7. Stability, Reproducibility, and Specificity of Dual-Mode Sensor

**Figure 6:**
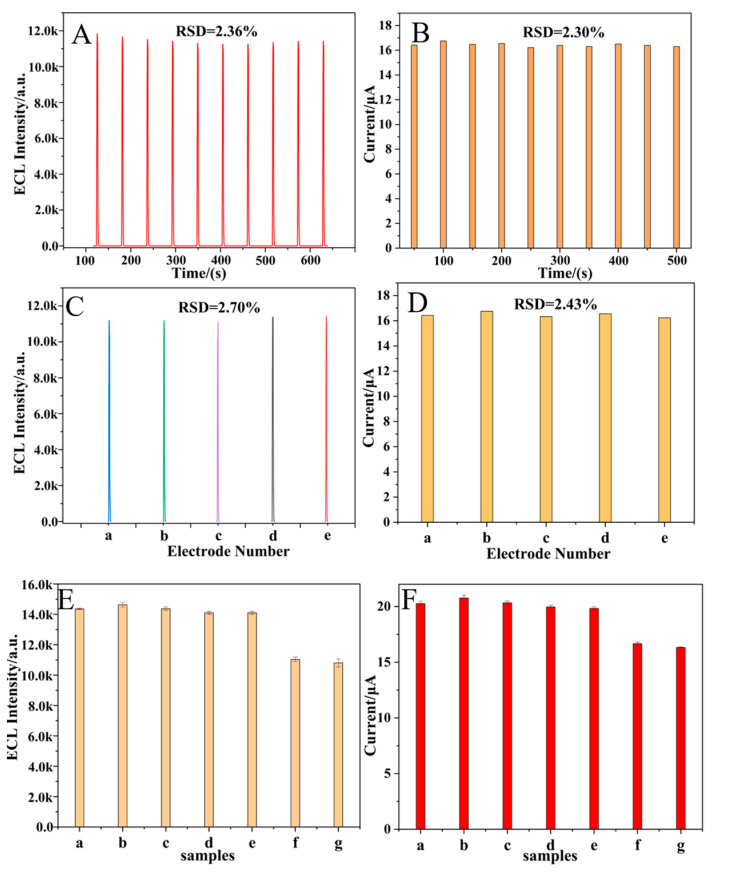
The stability of the dual-mode sensor’s ECL (**A**) and EC (**B**) signals for 500 nM Pb^2+^. The reproducibility of the dual-mode sensor’s ECL (**C**) and EC (**D**) signals for 500 nM/L Pb^2+^. The specificity of the dual-mode sensor’s ECL (**E**) and EC (**F**) signals from blank (a), 50 μM Ba^2+^ (b), 50 μM Mg^2+^ (c), 50 μM Zn^2+^ (d), 50 μM Ca^2+^ (e), 500 nM Pb^2+^ (f), and the mixture (g) (including all the above interfering substances). All average ECL intensities were obtained from three experimental results.

The stability, reproducibility, and specificity were important to evaluate the analytical performance of a sensor, so they were all studied in this work. As can be seen in Figure 6, the dual-mode sensor exhibited satisfactory stability over 10 consecutive scans, good reproducibility across five independent measurements, and excellent specificity in mixed solution containing Pb^2+^ and several interfering species.

### 2.8. The Practical Applicability of the Sensor

To further assess the practical applicability of the sensor, a recovery experiment was performed in water samples using the standard addition method. As shown in Table 3, the spiked recoveries ranged from 95.5 to 103.1%, with a relative standard deviation (RSD) of less than 8.0%. These results suggest that the dual-mode sensor developed in this study holds significant promise for the detection of Pb^2+^ in environmental samples.

## 3. Materials and Methods

### 3.1. Chemicals

The graphene oxide dispersion (GO) was purchased from Nanjing XFNano Materials Technology Co., Ltd. (Nanjing, China) Tris(2,2′-bipyridyl)ruthenium(II) chloride hexahydrate (Ru(bpy)_3_^2+^), hemin (hemin), and ascorbic acid (AA) were obtained from Alfa Aesar. Sodium oxalate (Na_2_C_2_O_4_), sodium hydroxide (NaOH), disodium hydrogen phosphate dihydrate (NaH_2_PO_4_·2H_2_O), disodium phosphate dodecahydrate (Na_2_HPO_4_·12H_2_O), sodium borohydride (NaBH_4_), sodium chloride (NaCl), barium chloride (BaCl_2_), calcium chloride (CaCl_2_), magnesium chloride (MgCl_2_), and zinc chloride (ZnCl_2_) were purchased from Tianjin Damao Chemical Reagent Factory. A 0.1 M phosphate-buffered solution (PBS) containing 100 mM NaCl and 10 mM KCl was prepared as the electrolyte for testing. The strand A15T5-G4 (3′-5′: AAA AAA AAA AAA AAA TTT TTG GGT AGG GCG GGT TGG GTC ATA CCA GCT TAT TCA ATT) was obtained from Shanghai Sangon Biotech Co., Ltd. (Shanghai, China), and dissolved in 0.1 M PBS. All solutions were prepared using deionized Millipore Milli-Q water (>18.2 MΩ·cm) from Sigma Aldrich Biotech Ltd (Wuxi, China).

### 3.2. Instruments

ECL measurements and cyclic voltammetry (CV) were carried out on a Model RFL−1 ECL analyzer (Xi’an Remex Instrument Co., Ltd., Xi’an, China) together with the auxiliary equipment of the CHI 900B electrochemical workstation (Shanghai CH Instruments Co., Shanghai, China). The voltage of the photomultiplier tube (PMT) was set at 650 V, and the scan rate was 0.1 V/s. Electrochemical impedance spectroscopy (EIS) was performed using a CHI 660E electrochemical workstation. (Shanghai CH Instruments Co., Shanghai, China). The CV scan rate was 0.1 V/s, and the frequency of EIS was from 0.01 to 10^5^ Hz. Scanning electron microscopy (SEM) and elemental mapping were performed using a Thermo Fisher Scientific FIB-SEM GX4 (Oberkochen, Germany). The experimental process was carried out using a classic three-electrode system, with a Ag/AgCl reference electrode (3 M KCl), a platinum wire counter electrode, and a modified glassy carbon working electrode (GCE, 3 mm, 0.07 cm^2^).

### 3.3. Fabrication of ECL/EC Dual-Mode Sensor

To ensure the smooth execution of the experiments, the GCE was first pretreated. Initially, the electrode was polished sequentially using 0.3 and 0.05 μm alumina powder. Following polishing, the electrode was ultrasonicated in ethanol and rinsed with water three times to remove any residual alumina particles and then dried using a nitrogen stream. The electrode’s performance was evaluated by conducting cyclic voltammetry in 1.0 mM K_4_[Fe(CN)_6_] over a potential range of −0.2 to 0.6 V at a scan rate of 50 mV/s, ensuring that the peak potential separation was below 78 mV. The polished electrode was stored in a refrigerator for subsequent use. Initially, water-soluble rGO was synthesized via a chemical reduction method [28]. Subsequently, 6 μL of 0.25 mg/mL of rGO solution was drop-cast onto a clean GCE surface and allowed to dry naturally at room temperature. Then, 6 μL of a 100 nM A15T5-G4 solution was incubated for 1 h at 37 °C to form GCE/rGO/A15T5-G4 through π–π stacking interactions between A15T5 and rGO. Thereafter, 6 μL of 50 μM hemin solution was added and incubated at 37 °C for 1.5 h to yield the GCE/rGO/G4–hemin. Finally, 6 μL of Pb^2+^ solution with different concentrations was incubated at 37 °C for 1 h, followed by collecting the ECL and EC signals. After each fabrication step, the electrode was rinsed with 0.1 M phosphate-buffered saline (PBS, pH 7.4). The fully assembled sensor was then stored at 4 °C for subsequent experiments.

### 3.4. Measurement Procedure

The developed sensor was immersed in 10 mM PBS including 0.35 mM Ru(bpy)_3_^2+^ and 0.5 mM Na_2_C_2_O_4_. Then, the ECL and EC signals were recorded during a one-step scan from 0.2 to −0.4 V with a scan rate of 0.1 V s^−1^.

## 4. Conclusions

In this study, a dual-mode ECL/EC sensor was developed based on rGO as the substrate and an A15T5G4–hemin composite structure as the catalyst. Pb^2+^ can weaken the binding affinity between G4 and hemin, leading to the release of hemin from the electrode surface and subsequently decreasing the electrochemical activity of the G4–hemin complex in the ORR. As a result, both ECL and EC signal intensities decrease synchronously at −0.25 V (vs. Ag/AgCl), enabling the one-step dual-mode detection of Pb^2+^ during a single potential scan from 0.2 to −0.4 V. Compared with previous studies, A15T5G4 can immobilize a greater amount of hemin, forming a more stable composite structure that significantly enhances both ECL and EC signal intensities, thereby improving sensor sensitivity and lowering the detection limit. Moreover, the introduction of this composite simplifies electrode modification procedures and reduces the overall fabrication cost of the sensor. The developed sensor exhibits several key advantages. First, the A15T5G4–hemin composite shows high selectivity toward Pb^2+^, effectively minimizing interference from other metal ions. Secondly, the enhanced adsorption capacity of A15T5G4 allows for greater hemin loading, further boosting ECL and EC responses. As a result, the sensor achieves high sensitivity with detection limits of 1.51 and 2.03 nM for ECL and EC modes, respectively, and demonstrates a wide linear detection range from 5 to 100 μM. Notably, the mutually calibrated ECL and EC peak signals both appear at −0.25 V (vs. Ag/AgCl), ensuring consistent detection conditions and preventing potential-induced damage to biomolecules on the electrode surface. These factors collectively contribute to improved detection accuracy and reliability. In conclusion, the dual-mode sensor developed in this study provides an effective approach for the highly sensitive detection of Pb^2+^, offers new insights into dual-mode sensing strategies, and promotes the application of ECL technology in environmental monitoring.

## Figures and Tables

**Table 1:** Comparison of the dual-mode sensor with other detection sensors for the detection of Pb^2+^.

Detection Method	Linear Range (nM)	Detection Limit (nM)	Ref.
FL	100–6 × 10^5^	18	[35]
EC	300–3 × 10^3^	50	[36]
FL	400–1.6 × 10^3^	450	[37]
ELISA	20–1 × 10^6^	200	[38]
SERS	50–1 × 10^3^	20	[39]
**ECL**	**5–1 × 10^5^**	**1.51**	**This work**
**EC**	**5–5 × 10^4^**	**2.03**	**This work**

**Table 2:** Comparison of the dual-mode sensor with previous assays.

Detection Method	ECL Potential Range (V)	EC Potential Range (V)	Ref.
ECL/EC	−2.0~0	−0.6~0.2	[41]
ECL/EC	−1.2~0	0~0.6	[42]
ECL/EC	0~1.5	−0.40~−0.8	[22]
ECL/EC	0~1.5	0~0.5	[43]
ECL/EC	1.0~1.8	−1.0~1.0	[23]
**ECL/EC**	**0.2~−0.4**	**0.2~−0.4**	**This work**

**Table 3:** Analysis of Pb^2+^ in environmental water sample.

Method	Added (nM)	Found (nM)	Recovery (%) (n = 3)	RSD % (n = 3)
ECL	0	10.13	-	0.60
	10.00	20.31	101.6	4.40
	100.0	105.3	95.51	3.60
	1000	993.4	99.44	7.10
EC	0	9.978	-	1.23
	10.00	20.47	103.1	4.78
	100.0	108.8	98.69	2.24
	1000	997.8	99.63	1.31

## Data Availability

The data that support the findings of this study are available from the corresponding author upon reasonable request.
